# Janus Nanoparticles in Doxorubicin Delivery: A New Frontier in Targeted Cancer Treatment

**DOI:** 10.3390/ma19081664

**Published:** 2026-04-21

**Authors:** Valeria Flores, Moniellen Pires Monteiro, Tanya Plaza, Jacobo Hernandez-Montelongo

**Affiliations:** 1 Departamento de Bioingeniería Traslacional, Centro Universitario de Ciencias Exactas e Ingenierías (CUCEI), Universidad de Guadalajara, Guadalajara 44430, Mexico; 2 Departamento de Física, Universidad Técnica Federico Santa María, Valparaíso 2390123, Chile; moniellen.pires@usm.cl; 3 Departamento de Ciencias Físicas y Matemáticas, Universidad Católica de Temuco, Temuco 4813302, Chile; tplaza@uct.cl; 4 Núcleo de Investigación en Bioproductos y Materiales Avanzados (BioMA), Universidad Católica de Temuco, Temuco 4813302, Chile

**Keywords:** Janus nanoparticles, doxorubicin, cancer treatment, drug delivery

## Abstract

Cancer remains a primary global health challenge, accounting for millions of new cases and significant mortality annually. Although doxorubicin (DOX) is a fundamental anthracycline used for various malignancies, its therapeutic index is severely limited by poor selectivity, systemic toxicity, and dose-dependent cardiotoxicity. To address these issues, Janus nanoparticles (JNPs) have emerged as a promising bifunctional platform characterized by a structural asymmetry that allows for the independent functionalization of each hemisphere. This review examines primary fabrication routes—such as masking, microfluidics, self-assembly, and phase separation—and their specific applications in DOX delivery. The anisotropic architecture of JNPs enables a “separate rooms” concept, allowing for the co-delivery of incompatible drugs while facilitating multi-stimuli-responsive release mechanisms triggered by pH, enzymes, or NIR light. Furthermore, JNPs have demonstrated enhanced tumor accumulation and reduced systemic toxicity compared to conventional isotropic carriers. Recent developments even highlight the use of autonomous nanomotors to improve therapeutic delivery while minimizing premature leakage. However, clinical translation is currently hindered by manufacturing complexity, high equipment costs, scalability issues, and a lack of standardized reporting in the literature. Ultimately, JNPs represent a sophisticated frontier in precision oncology, though robust manufacturing processes and characterization protocols are required for future medical adoption.

## 1. Introduction

Cancer remains one of the leading causes of mortality worldwide, with millions of new cases diagnosed annually [1]. The global burden of the disease is considerable; according to the Global Burden of Disease (GBD) 2023 study, cancers accounted for approximately 271 million disability-adjusted life years (DALYs), encompassing both premature mortality (years of life lost, YLL) and disease-related disability (years lived with disability, YLD) [2]. Despite significant advancements in treatment strategies, conventional chemotherapy remains a primary approach. However, traditional chemotherapeutic agents, such as doxorubicin (DOX), suffer from major drawbacks, including poor selectivity and severe systemic toxicity, leading to apoptosis and necrosis in healthy tissues. This off-target damage affects vital organs such as the brain, liver, kidneys, and heart, significantly reducing DOX’s therapeutic efficacy [3,4]. In particular, DOX—an anthracycline widely used in the treatment of breast cancer, leukemia, and lymphoma—is strongly associated with dose-dependent cardiotoxicity, which limits its clinical application [5,6].

To address these challenges, nanotechnology-based drug delivery systems have been developed to enhance targeted drug delivery, optimize controlled release, and improve biocompatibility [7]. Among these, Janus nanoparticles (JNPs) have emerged as a particularly promising platform due to their dual-functional properties, enabling site-specific drug delivery while minimizing systemic toxicity [8,9].

**Figure 1:**
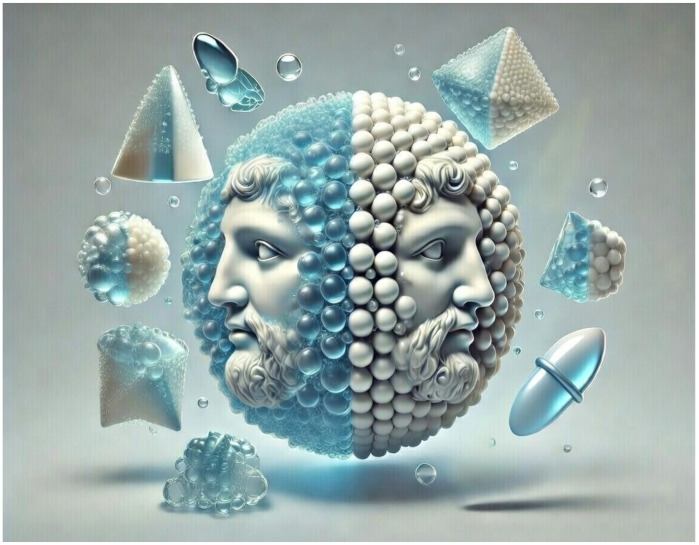
Conceptual illustration of Janus nanoparticles highlighting asymmetric surface domains and morphological configurations.

Named after the two-faced Roman god Janus, JNPs feature an asymmetrical structure, allowing for distinct functionalization on each side (Figure 1). Unlike conventional spherical nanoparticles, JNPs are designed to integrate two or more unique properties within a single nanoparticle, enabling simultaneous execution of multiple functions [9,10]. This bifunctional nature makes them highly suitable for stimuli-responsive drug release, combination therapy, and theranostics [8,11].

The structural asymmetry of JNPs confers clear advantages over conventional nanocarriers in targeted drug delivery. Their compartmentalized architecture enables the simultaneous loading of hydrophilic and hydrophobic drugs, with loading efficiencies reaching up to ∼92% in optimized systems [12,13], often matching or exceeding those of traditional carriers [14,15]. In addition, surface functionalization with targeting ligands enhances selectivity, leading to up to a 4-fold reduction in ${\mathrm{IC}}_{50}$ [16] and up to a 15-fold increase in intracellular drug accumulation under externally triggered conditions [17], outperforming non-targeted and conventional systems [18,19].

JNPs also enable stimuli-responsive drug release (e.g., pH, temperature, or enzymatic triggers), achieving 70–94% release under tumor-relevant conditions [13,20], in contrast to the burst-release behavior typical of diffusion-controlled nanocarriers [9,21]. These properties translate into enhanced therapeutic performance, with tumor suppression rates approaching ∼96% in in vivo models [13,22]. Beyond delivery, JNPs support theranostic applications, enabling real-time monitoring of biodistribution and treatment response [19,23]. However, direct quantitative comparisons across systems remain limited due to variability in experimental design and evaluation metrics.

By leveraging their multifunctional properties, JNPs provide versatile strategies to overcome key limitations of conventional chemotherapy, particularly in DOX delivery. For instance, magnetic JNPs have been employed as dual-function DOX carriers and therapeutic agents, enabling combined chemotherapy and magnetic hyperthermia under MRI guidance [24,25]. Similarly, pH-responsive JNPs selectively release DOX in acidic tumor environments, minimizing premature leakage [25,26,27], while lipid–polymer hybrid JNPs extend circulation time and enhance cellular uptake compared to conventional liposomal formulations [28].

In this context, the present review provides a comprehensive overview of JNPs as an emerging platform for DOX delivery in cancer therapy. It addresses cancer as a global health challenge, examines the role of DOX as a versatile chemotherapeutic agent, and discusses the synthesis and physicochemical characterization of JNPs. Furthermore, it critically surveys recent advances in JNP-based DOX delivery systems and outlines current limitations and future perspectives, highlighting their potential to advance precision oncology and clinical translation.

## 2. Materials and Methods

### 2.1. Literature Search

This review focused on JNPs as an emerging platform for DOX delivery in cancer therapy. A systematic literature search was conducted in 2025 across the major databases: Web of Science, Scopus, and PubMed. Supplementary searches were performed using ResearchGate and Google Scholar to ensure comprehensive coverage.

### 2.2. Generative Artificial Intelligence Tools

Generative Artificial Intelligence (GenAI) tools were used in a limited, supportive capacity during the preparation of this review. ChatGPT Plus (GPT-5.3) and Gemini 3 Flash were employed to assist in identifying potentially relevant literature and suggesting references on specific topics. NotebookLM was used to generate initial summaries of selected publications and to facilitate extraction of key data. For language refinement, ChatGPT Plus (GPT-5.3) and Claude 4.6 Opus were utilized to improve grammar, clarity, and readability. Additionally, NotebookLM and Grok 4 assisted in improving the presentation and clarity of figures. The final selection, screening, and critical appraisal of all articles, as well as verification of summaries and extracted data against original sources, were performed independently and exclusively by the authors. All scientific interpretations, analyses, conclusions, and the overall intellectual content of this review are the sole responsibility of the authors. All outputs generated by GenAI tools were critically reviewed, edited, and finalized by the authors. At no stage did GenAI tools replace human scientific judgment.

## 3. Cancer: A Global Health Challenge

Cancer encompasses a diverse group of diseases characterized by the uncontrolled proliferation and dissemination of abnormal cells within the body. Under normal physiological conditions, cells grow, divide, and undergo apoptosis in a tightly regulated manner. However, cancer cells circumvent these control mechanisms, leading to persistent and unregulated growth, often resulting in the formation of solid masses known as tumors [29]. Tumors are classified as either benign (non-cancerous) or malignant (cancerous), with malignant tumors capable of invading surrounding tissues and metastasizing to distant organs through the bloodstream or lymphatic system [30,31].

The initiation of cancer is primarily driven by genetic mutations arising from hereditary factors, environmental exposures (e.g., radiation and carcinogens), and lifestyle-related influences such as tobacco use, poor diet, and physical inactivity [32,33,34]. Although cells possess intrinsic DNA repair and apoptotic mechanisms to maintain genomic integrity, the failure of these systems enables the uncontrolled proliferation of damaged cells [35,36]. Carcinogenesis is therefore understood as a multistep process involving the progressive accumulation of genetic alterations, including the activation of oncogenes and the inactivation of tumor suppressor genes, ultimately leading to dysregulated cell cycle control and tumor development [37]. In recent years, increasing attention has been directed toward advanced biophysical techniques capable of capturing early phenotypic alterations associated with these molecular events. In this context, atomic force microscopy (AFM) has emerged as a powerful tool for nanoscale characterization of cellular surfaces, providing mechanical and topographical information that complements conventional molecular approaches. Notably, the integration of AFM with machine learning strategies has further enhanced its potential, enabling ultrasensitive detection and classification of early-stage cancer-related changes [38,39].

In 2022 alone, cancer was responsible for nearly 10 million deaths worldwide. Lung cancer was the leading cause of cancer-related mortality, accounting for approximately 1.8 million deaths (18.7%), followed by colorectal cancer (9.3%), liver cancer (7.8%) and, stomach cancer and female breast cancer (6.8%) [40]. This substantial mortality burden is further reflected in the high number of disability-adjusted life years (DALYs), with approximately 271 million attributed to cancer globally in 2023, highlighting the urgent need for therapeutic strategies that reduce both toxicity and long-term disease impact [2].

According to estimates from the Global Cancer Observatory (GCO), based on the International Statistical Classification of Diseases and Related Health Problems (ICD-10), approximately 21.3 million new cancer cases were diagnosed worldwide in 2025. This figure represents a 6.8% increase compared with 2022 [40]. Moreover, the global cancer burden is projected to increase substantially, with an estimated 29.9 million new cases by 2040—corresponding to a 49.6% rise relative to 2022—assuming constant national incidence rates [40]. The most frequently reported cancer types in 2025 are summarized in Table 1.

## 4. Doxorubicin: A Versatile Drug in Oncology

**Figure 2:**
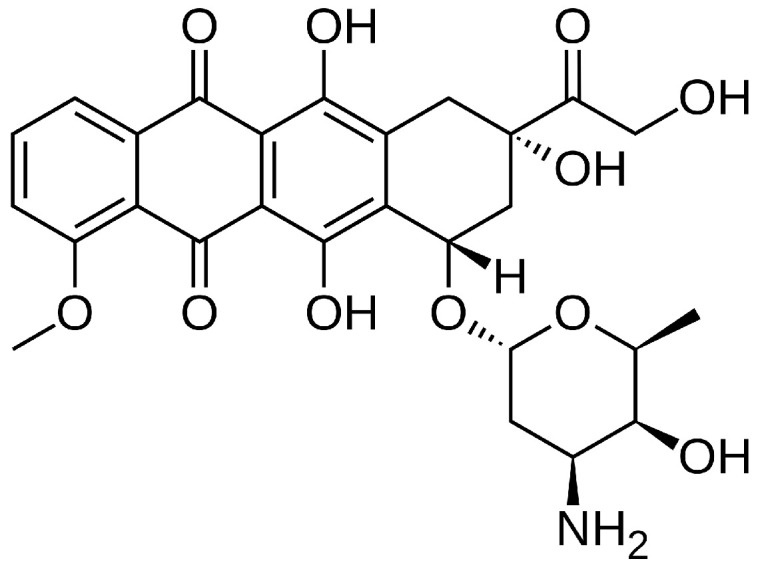
Chemical structure of doxorubicin (DOX) ($C_{27}H_{29}{\mathrm{NO}}_{11}$; molecular weight: 543.52 g/mol).

The high mortality associated with cancer highlights the ongoing need for research into improved treatments and prevention strategies. A central challenge in cancer therapy is achieving a balance between therapeutic efficacy and minimizing adverse side effects [41]. DOX, a widely used chemotherapeutic agent, exemplifies both the clinical potential and limitations encountered in modern oncology (Figure 2) [42].

DOX is an anthracycline antibiotic discovered in the 1960s and remains a cornerstone of chemotherapy protocols due to its broad-spectrum activity against numerous malignancies, including bladder, breast, stomach, lung, ovarian cancers, and Hodgkin’s lymphoma [42,43]. It was originally isolated from *Streptomyces peucetius*, a bacterium that also produces daunorubicin, a closely related compound. Through genetic manipulation, DOX—commercially known as Adriamycin—was developed to exhibit a higher therapeutic index [42]. Despite its potent anticancer activity, its clinical utility is significantly constrained by dose-dependent cardiotoxicity, which limits both dosage and duration of treatment [44,45,46].

**Figure 3:**
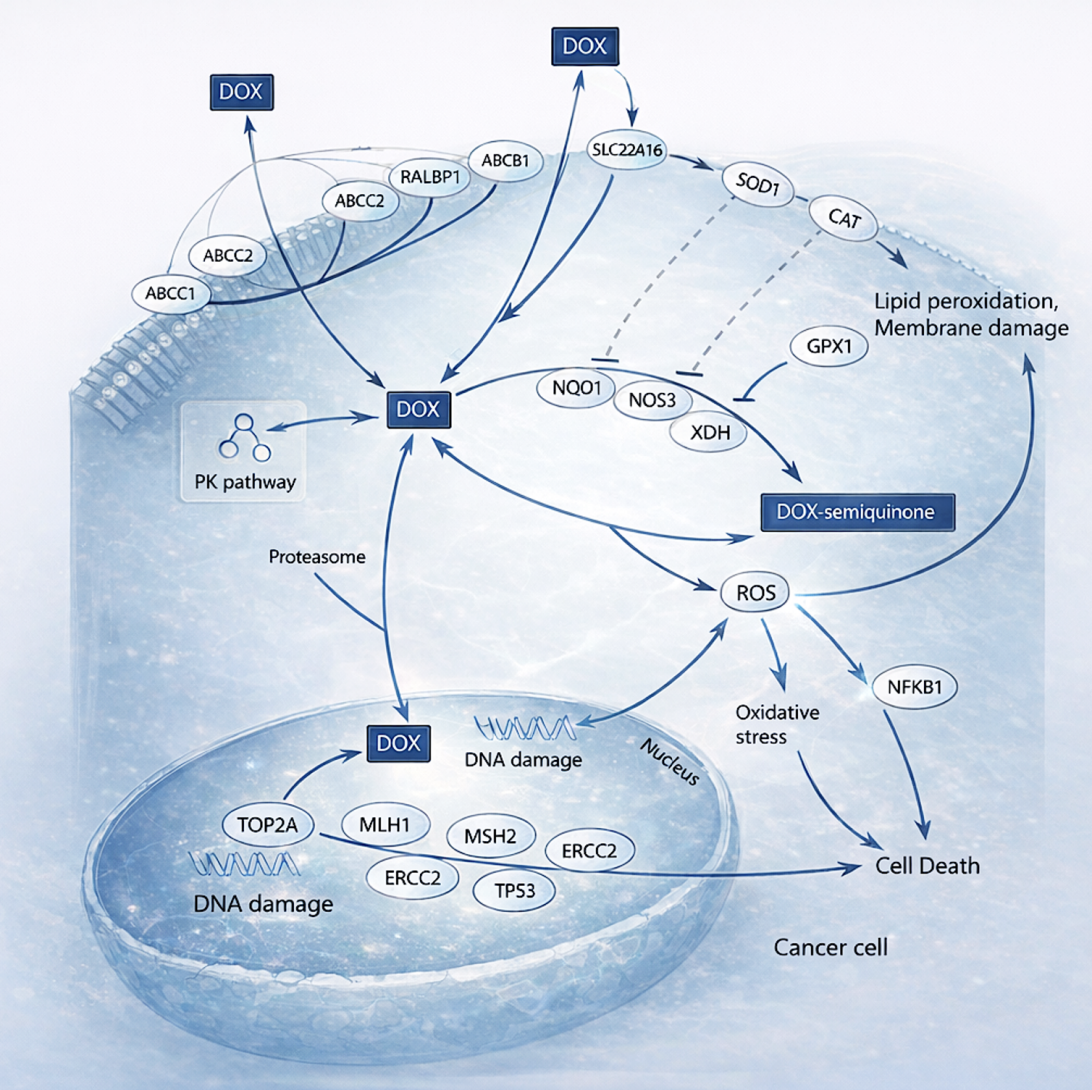
Mechanism of action of doxorubicin (DOX) against cancer cells. Adapted from Rivankar S., (2014) [42].

Figure 3 illustrate the mechanism of action of DOX, which is multifaceted, contributing to its effectiveness in cancer therapy [42]. Primarily, it intercalates into DNA, inserting between base pairs and disrupting the helical structure. This interference impairs the synthesis of macromolecules essential for cell proliferation [47]. In addition, doxorubicin inhibits topoisomerase II, an enzyme critical for DNA replication and transcription [48]. By stabilizing the cleaved DNA–topoisomerase II complex, it prevents the re-ligation of DNA strands, thereby halting these vital cellular processes and promoting apoptosis in rapidly dividing cancer cells [42].

Moreover, DOX induces oxidative stress through the generation of free radicals during its metabolic interaction with cellular components. These reactive species inflict further cytotoxic damage to DNA, proteins, and lipid membranes, enhancing its anticancer effects [46,49]. However, these same free radicals are also implicated in the cardiotoxic effects observed with doxorubicin therapy, damaging myocardial tissue and significantly restricting its long-term use [46,49,50].

To address these limitations, alternative formulations have been developed to reduce systemic toxicity. Liposomal encapsulation of doxorubicin aims to protect healthy tissues—especially the heart—by altering its biodistribution and improving tumor selectivity [51]. While such formulations show promise in minimizing adverse effects, they often require complex administration protocols, presenting additional challenges in clinical practice [42].

## 5. Pharmacokinetics of Doxorubicin

**Figure 4:**
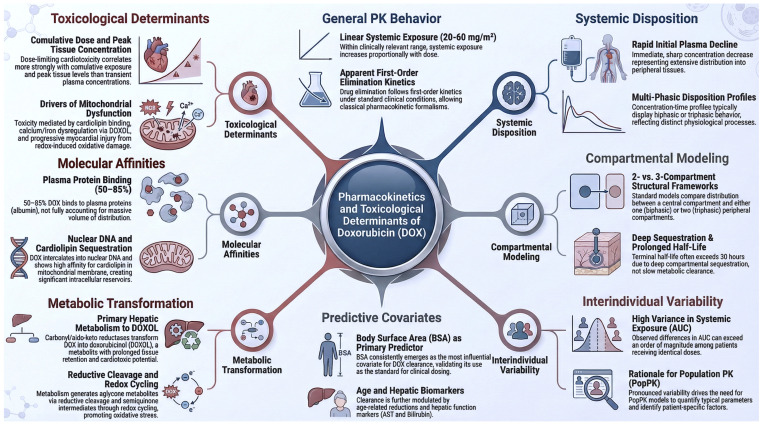
Pharmacokinetics and toxicological determinants of doxorubicin (DOX). The schematic begins with the General PK Behavior section and proceeds clockwise.

This section addresses the pharmacokinetics of DOX, from its systemic disposition to its toxicological determinants, describing how the drug is distributed, metabolized, and eliminated within the body. An overview of these processes is schematically illustrated in Figure 4.

### 5.1. General Pharmacokinetic Behaviour and Kinetic Order

Within the clinically relevant dose range (approximately 20–60 mg/m^2^), DOX exhibits predominantly apparent linear pharmacokinetics in population-averaged analyses, with systemic exposure increasing proportionally with dose [52,53]. Under standard clinical conditions, drug elimination follows apparent first-order kinetics, allowing the use of classical pharmacokinetic formalisms for describing plasma concentration–time profiles [54].

However, this apparent linearity should not be interpreted as pharmacokinetic simplicity, nor as a universal constant across all patient demographics. Rather, it reflects an averaged systemic behaviour that masks extensive tissue distribution, deep compartmental sequestration, and prolonged terminal elimination phases [54], along with significant inter-individual variability and potential non-linearities linked to sex, age, and ancestral genetic background [55]. These demographic variables, which influence drug metabolism and tissue distribution, necessitate more complex structural models to adequately describe the disposition of DOX in vivo, as discussed in Section 5.4 and Section 5.5.

### 5.2. Systemic Disposition and Distribution Phases

Following intravenous administration, DOX undergoes a rapid initial decline in plasma concentration, corresponding to an extensive and fast distribution into peripheral tissues. This early phase is typically followed by one or more slower phases reflecting redistribution and elimination processes [54,56].

Clinically observed concentration–time profiles therefore display either biphasic or triphasic behaviour, depending on study design, sampling duration, and infusion protocol. These phases are not merely mathematical artefacts but represent distinct physiological processes, including vascular distribution, tissue binding, intracellular sequestration, and slow release from deep tissue reservoirs [56].

### 5.3. Classical Compartmental Models: Two- and Three-Compartment Structures

To formalise these observations, the pharmacokinetics of DOX are most commonly described using multi-compartment models [57]. Two- and three-compartment models constitute the standard structural frameworks employed in both individual and population-based analyses [54,57,58].

In two-compartment models, the drug distributes between a central compartment (plasma and highly perfused tissues) and a single peripheral compartment, resulting in a biphasic disposition profile. In contrast, three-compartment models incorporate an additional deep peripheral compartment, capturing a rapid distribution phase, an intermediate redistribution phase, and a slow terminal elimination phase [54,57].

The long terminal half-life of DOX, often exceeding 30 h, is a direct consequence of this deep compartmental sequestration rather than slow metabolic clearance alone [59].

### 5.4. Interindividual Variability and the Rationale for Population Pharmacokinetic Modelling

Despite the adequacy of these models in describing mean pharmacokinetic behaviour, DOX is characterised by exceptionally high interindividual variability [52,53]. Differences in systemic exposure, commonly quantified by the area under the concentration–time curve (AUC), may exceed an order of magnitude between patients receiving identical doses [58].

This pronounced variability has driven the development of population pharmacokinetic (PopPK) models, which aim to quantify both typical parameter values and their variability within a population [57]. These models incorporate random effects to describe unexplained variability and provide a statistical framework for identifying patient-specific factors that influence drug disposition [58].

### 5.5. Predictive Covariates in Population Models

A central objective of PopPK modelling is the identification of covariates that explain variability in key pharmacokinetic parameters, particularly clearance and volume of distribution [57,59]. Among demographic predictors, body surface area (BSA) has consistently emerged as the most influential covariate for DOX clearance, supporting the long-standing clinical practice of BSA-based dosing [57,58].

Beyond BSA, biological sex is a key yet often overlooked determinant of doxorubicin pharmacokinetics. Sex-related differences arise from hormonal modulation of hepatic cytochrome P450 enzymes (e.g., CYP3A4, CYP2D6), affecting drug clearance and metabolite formation, with menopause further altering these dynamics over time [60,61,62]. Age also contributes to variability, with reduced clearance observed in adult and elderly populations, often accompanied by changes in body composition that influence drug distribution [57,58].

Genetic variability across populations, particularly in pharmacogenes such as CYP3A4, CYP2D6, and UGT, further contributes to inter-individual differences in metabolism, while disparities in healthcare access may impact toxicity outcomes [63,64]. Physiological factors, including hepatic function (e.g., AST, bilirubin), renal function, and pregnancy, also play a critical role in modulating pharmacokinetics [65,66].

Together, these covariates highlight the limitations of BSA-based dosing and support the need for biologically informed, model-based approaches to enable precision-guided therapy.

### 5.6. Metabolism of Doxorubicin: Major Metabolites and Mechanisms of Formation

The systemic clearance of DOX is largely governed by extensive hepatic metabolism, although extrahepatic metabolism in the kidneys and erythrocytes also contributes [59]. Metabolic transformation plays a dual role: facilitating elimination while simultaneously generating metabolites with distinct pharmacological and toxicological properties [65]. The relevance of metabolism therefore extends beyond pharmacokinetics into the domain of drug-induced toxicity, particularly cardiotoxicity [52,53,67].

The principal metabolite of DOX is doxorubicinol (DOXOL), formed via reduction of the carbonyl group by carbonyl reductases (CBR1 and CBR3) and aldo-keto reductases. Although DOXOL is less potent as an antitumour agent than the parent compound, it exhibits prolonged tissue retention and is strongly implicated in cardiac dysfunction [68].

In addition to DOXOL, reductive cleavage of the daunosamine sugar moiety generates aglycone metabolites, such as doxorubicinone and 7-deoxydoxorubicinone. These species lack antineoplastic activity but have been associated with myocardial injury. In addition, redox cycling of the quinone moiety further produces unstable semiquinone intermediates, which generate reactive oxygen species upon re-oxidation, amplifying oxidative stress within susceptible tissues [68].

### 5.7. Plasma Protein Binding and Tissue Affinity

In systemic circulation, approximately 50–85% of DOX is bound to plasma proteins, predominantly albumin. However, protein binding alone does not account for the exceptionally large apparent volume of distribution, which often exceeds 20–30 L/kg [54].

Tissue distribution is instead dominated by high-affinity interactions with intracellular macromolecules. DOX intercalates extensively into nuclear DNA, effectively rendering the nucleus a major intracellular drug reservoir [52,53]. Additionally, the drug displays a strong affinity for cardiolipin, a phospholipid enriched in the inner mitochondrial membrane, particularly within cardiomyocytes [59,67].

These interactions govern both tissue accumulation and prolonged retention, decoupling plasma concentrations from intracellular exposure.

### 5.8. Tissue Affinity, Cardiotoxicity, and Systemic Toxicity

The preferential accumulation of DOX and its metabolites in cardiac tissue provides a mechanistic basis for its dose-limiting cardiotoxicity [69]. Binding to cardiolipin disrupts mitochondrial function [67], while DOXOL exacerbates calcium and iron dysregulation within cardiomyocytes. Concurrently, redox cycling promotes oxidative damage, leading to progressive myocardial injury [65,68].

Importantly, cardiotoxicity correlates more strongly with cumulative dose and peak tissue concentrations than with plasma levels alone [54]. This dissociation underscores the limitations of conventional pharmacokinetic metrics and highlights the need for strategies that modulate tissue exposure rather than systemic dose [67,69].

In this context, the complex pharmacokinetic and tissue distribution profile of DOX represents a fundamental challenge that has directly motivated the development of advanced drug delivery systems, including nanostructured and Janus-based formulations.

## 6. Janus Nanoparticles as Out-of-Equilibrium Matter

Micro- and nanometric Janus particles are characterized by an intrinsic asymmetry that endows them with direction-dependent physical and chemical properties. Owing to their distinctive hemispherical architecture, these particles can decouple physicochemical functionalities within a single entity, for example by simultaneously exhibiting hydrophobic and hydrophilic domains or integrating separate sensing, targeting, and labeling regions. This structural versatility has positioned JNPs as a powerful materials platform for applications in bionanotechnology and nanomedicine [70,71].

For biomedical applications, the biodistribution of JNPs is a key determinant of their in vivo performance and biosafety. Their fate in biological systems is governed by physicochemical parameters—such as composition, size, surface chemistry, and functionalization—as well as the route of administration, which collectively influence circulation time, tissue accumulation, and biological interactions. In cancer therapy, JNPs can preferentially accumulate in tumors through passive targeting via the enhanced permeability and retention (EPR) effect, while active targeting further enhances selectivity through surface functionalization with ligands such as folic acid, lactobionic acid, or receptor-specific proteins (e.g., ZHER2:342). Notably, the intrinsic asymmetry of JNPs allows the spatial separation of targeting moieties and therapeutic agents, improving delivery efficiency and therapeutic precision. In addition, polyethylene glycol (PEG) modification is widely used to enhance colloidal stability, minimize nonspecific protein adsorption, and optimize biodistribution and clearance profiles [23]. Together, these design strategies contribute to improved biosafety, as JNPs based on biocompatible materials have demonstrated low cytotoxicity, enhanced therapeutic selectivity, and reduced systemic toxicity compared to conventional chemotherapeutics such as DOX [7,8,9,11].

Compared with conventional nanoparticle-based carriers, JNPs offer distinct advantages for DOX delivery by enabling spatial separation of drug payloads and functional domains within a single carrier. While isotropic systems such as liposomes and polymeric nanoparticles have demonstrated clinical success in reducing systemic toxicity and improving drug tolerability [3,7], they often suffer from burst release and limited control over combination therapies [14]. In contrast, Janus architectures allow DOX to be confined to a dedicated compartment, while the opposing domain can be independently engineered for targeting, imaging, or secondary therapeutic functions [9,10]. This anisotropic design facilitates multi-stimuli-responsive release and improved synchronization of pharmacokinetics in combination treatments, addressing key limitations of conventional DOX nanocarriers [15,21]. Moreover, JNPs have been reported to reduce macrophage uptake and prolong circulation persistence due to shape- and interface-driven effects, thereby enhancing tumor accumulation [8,23].

A comparative summary of these differences in DOX release behavior, advantages, and limitations between conventional nanoparticles and Janus nanoparticles is provided in Table 2. Despite these advantages, the clinical translation of Janus DOX delivery systems remains limited by fabrication complexity, scalability, and regulatory challenges [10,11].

Beyond drug delivery, JNPs are also paradigmatic systems in the field of active matter. Their motility originates from intrinsic structural and chemical asymmetry, which enables the conversion of environmental energy into persistent motion. In chemically driven systems, propulsion typically arises from self-phoretic mechanisms [72,73], whereby asymmetric surface reactions generate local gradients in concentration, electric potential, or interfacial tension that induce directed motion [74]. While the propulsion direction remains fixed relative to the functionalized hemisphere, rotational diffusion progressively randomizes particle orientation, leading to a crossover from ballistic motion at short timescales to enhanced diffusive behavior at longer times [75]. External magnetic or electric fields can further modulate particle motion and orientation, providing additional control over JNPs dynamics [73,74].

At higher particle densities or activity levels, interactions between motile JNPs give rise to collective nonequilibrium phenomena, including dynamic clustering, swarming, and motility-induced phase separation. These behaviors emerge from the interplay of persistent self-propulsion with steric, hydrodynamic, and phoretic interactions, often mediated by self-generated chemical or thermal fields. Such collective states establish JNPs systems as model platforms for studying active matter, offering experimental access to nonequilibrium phase behavior, collective motion, and emergent order at the microscale [76,77].

### 6.1. Janus Nanoparticles Fabrication Ways

**Figure 5:**
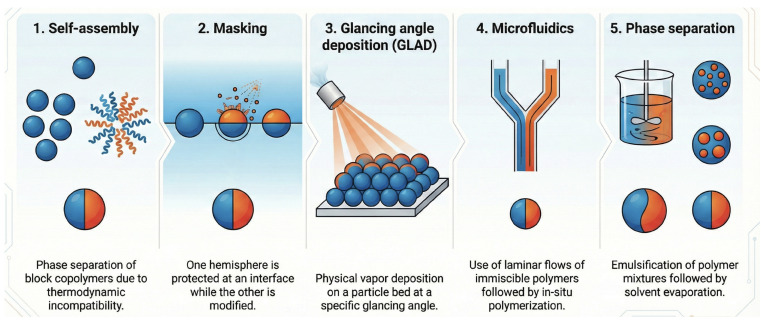
Primary methods for Janus nanoparticles (JNPs).

Over the past decades, significant effort has been devoted to the development of methods for generating Janus structures with controlled composition and structure. wide range of morphologies and particle geometries can be achieved in the synthesis of Janus nanoparticles (JNPs), which are commonly classified according to five principal fabrication routes: bottom-up self-assembly, surface-selective masking, glancing angle deposition (GLAD), microfluidic approaches, and phase-separation-driven processes (Table 3). Figure 5 provides a schematic overview of these fabrication strategies.

Bottom-up self-assembly methods typically yield low to moderate amounts of JNPs, but they are often limited by high aggregation and low to moderate monodispersity, since particle formation relies on spontaneous molecular organization that is difficult to precisely control [10,78]. Surface-selective masking approaches improve structural control, producing particles with moderate yields, low aggregation, and high monodispersity. This is because they begin with uniform precursor particles and selectively modify only one hemisphere, enabling consistent size and morphology [79,80]. GLAD further enhances anisotropic precision, resulting in low aggregation and high monodispersity, but at the expense of low yield due to the slow and equipment-intensive nature of physical vapor deposition techniques [81]. Microfluidic approaches provide a strong balance between control and productivity, offering moderate to high yields, low aggregation, and very high monodispersity. The precise manipulation of fluids at the microscale allows for highly uniform particle formation and minimal clustering [82]. Finally, phase-separation-driven processes are well suited for large-scale production, achieving high yields, but they generally exhibit moderate to high aggregation and low to moderate monodispersity, as the phase separation process is less controlled and leads to broader size distributions [83].

Early and commercially implemented approaches include the production of dual-colored microspheres for electrophorectic paper applications, which are formed through the fragmentation of stratified wax streams at the edge of a rotating disk. While this process is suitable for large-scale manufacturing, it offers limited control over particle monodispersity and frequently results in broad size distributions. A variety of alternative fabrication techniques—ranging from asymmetric metal deposition [84,85] and colloidal self-assembly [86] to microcontact-based patterning [87] and electrohydrodynamic jetting [88]—have been investigated to expand the library of Janus architectures. Nevertheless, challenges related to scalability and production throughput continue to limit the translation of many of these methods into widespread practical use.

#### 6.1.1. Self-Assembly

Self-assembly represented one of the first strategies explored for the generation of JNPs. In approaches based on block copolymers, asymmetric particle formation is dictated by the thermodynamic behavior of polymer mixtures and by the precise tuning of the conditions that regulate spontaneous organization. Parameters such as temperature and, in systems containing polyelectrolytes, the solution pH and ionic strength critically determine the development of compositional asymmetry. Despite its versatility, this methodology faces inherent challenges in scaling up, since controlled and uniform assembly becomes increasingly difficult at elevated polymer concentrations [89].

The coexistence of hydrophilic and hydrophobic regions within a single particle is a defining feature that underlies the broad interfacial functionality of Janus particles, as early conceptualized by de Gennes. Owing to their resemblance to molecular surfactants, JNPs have been described using the Janus balance (JB), a parameter introduced to quantify their relative affinity for oil and water. This dimensionless descriptor, derived from particle geometry and interfacial contact angles, provides a useful framework for anticipating the type of Pickering emulsion stabilized by a given Janus system. Experimental investigations employing silica-based Janus particles with tunable JB values have confirmed that systematic variations in particle wettability can drive emulsion phase inversion between oil-in-water and water-in-oil regimes [90].

#### 6.1.2. Masking

Polymeric JNPs can be fabricated through a variety of masking-based strategies. These approaches rely on the selective exposure of only part of a particle surface to a reactive environment, enabling localized chemical modification while the remaining surface is shielded from functionalization. Among the available fabrication routes, masking methods are often regarded as particularly versatile, as they are broadly compatible with diverse material systems and allow extensive control over surface chemistry. In practice, masking can be implemented by immobilizing polymer particles at fluid–fluid interfaces or by temporarily fixing them onto solid substrates, thereby defining spatially confined regions for asymmetric modification [89,91,92].

Interfaces between immiscible phases—such as air–liquid, liquid–liquid, or solid–liquid boundaries—were among the earliest platforms used to generate surface anisotropy in colloidal particles. These systems act as physical masks: one portion of a particle is shielded by the interface, while the exposed region undergoes chemical modification. Early demonstrations of amphiphilic JNPs relied on hemispherical protection of glass beads, followed by selective functionalization of the unprotected side to introduce hydrophobic chains. Since then, silanization of silica surfaces has remained a widely used strategy due to the synthetic versatility of organosilanes bearing diverse terminal groups.

Other surface-specific reactions can be implemented when particles contain reactive functionalities, enabling selective coupling—such as biomolecule attachment—on only the exposed domain. However, planar interfaces inherently restrict production because only a monolayer of particles can be modified at a time. The adoption of emulsion-based systems significantly increased interfacial area, allowing larger-scale synthesis through liquid–liquid masking [93].

Despite their simplicity, interface-mediated methods face limitations. The range of compatible chemistries may be narrow, spatial control over the modified region can be affected by interfacial fluctuations and reagent diffusion, and particle orientation is typically well-defined only for spherical geometries. Additionally, complete removal of masking materials and residual reagents remains an important practical consideration. Early demonstrations of masking-based fabrication routes for JNPs employed polystyrene microspheres as templates, onto which a thin gold layer was selectively deposited to generate surface asymmetry [94,95].

#### 6.1.3. Glancing Angle Deposition

Glancing angle deposition (GLAD) has become one of the most established fabrication routes for generating anisotropic colloids used in active matter systems. Since its development in the late 1990s, this vapor-phase technique has enabled the precise creation of surface asymmetry, a key structural requirement for self-propelled particles. The approach is based on physical vapor deposition or sputtering onto colloidal particles that have been pre-assembled into a dense monolayer on a substrate. Because material arrives from a directional vapor flux, only the exposed regions of each particle are coated, naturally producing spatially asymmetric surface functionalization.

When the particle monolayer is positioned perpendicular to the incoming vapor beam, deposition occurs preferentially on one hemisphere, yielding classical Janus architectures. Such half-coated particles are particularly relevant for active colloids, where the metallic cap often serves as a catalytic site (e.g., platinum-driven decomposition of hydrogen peroxide) or as a light-absorbing layer for photothermal propulsion. By systematically varying the tilt angle between the substrate and the vapor source, the extent of surface coverage can be adjusted, allowing fine control over patch size, geometry, and thus propulsion characteristics. This tunability is crucial, as propulsion speed, torque generation, and directional stability are strongly influenced by the spatial distribution of surface functionality.

Metallic coatings—most commonly platinum, titanium, or chromium—are frequently employed due to their catalytic, optical, or magnetic properties. However, carbonaceous and other functional thin films have also been integrated to enable alternative propulsion mechanisms or multi-responsive behavior. Although the chemistry of the deposited material must be compatible with vapor-phase processing, the core particle itself can be composed of a broad range of materials, provided that it can be assembled into a uniform monolayer prior to deposition.

From a practical perspective, scalability remains a significant engineering consideration. Traditional GLAD implementations are typically limited to wafer-scale substrates, which constrains production throughput. Nevertheless, these limitations stem primarily from current processing formats rather than fundamental physical constraints. The integration of large-area or continuous monolayer assembly with scalable vapor deposition platforms could, in principle, enable higher-yield production of active colloids for collective behavior studies and emerging applications [96,97].

#### 6.1.4. Microfluidics

The earliest reports of this fabrication method for spherical Janus particles date back to 2007, when Nisisako et al. employed a microfluidic system with Y-shaped channels to generate biphasic Janus droplets [98]. Biphasic droplet formation was achieved using a photocurable polymer phase and an immiscible silicone oil phase. Each fluid was independently injected through one branch of a Y-shaped microfluidic junction, where the two dispersed streams merged and were subsequently segmented by a continuous external phase. Droplet breakup was governed by shear forces at the junction, and the droplet size and monodispersity were finely tuned through precise regulation of the flow rates in all three channels, resulting in the reproducible generation of uniformly sized biphasic droplets. Biphasic droplet formation is governed by interfacial energy minimization, making precise control of interfacial tensions among the participating liquid phases essential for tuning particle morphology. Adjusting the type and concentration of surfactants in the aqueous phase influences droplet shape, while the incorporation of lipophilic surfactants in the organic phases modulates the extent of droplet constriction.

As an applied example of Janus particles fabricated using microfluidic devices, Sundararajan et al. [70] reported the use of poly(lactic-co-glycolic acid) (PLGA) as a biocompatible polymer matrix and titanium dioxide as a model cargo. In this system, the active component is selectively localized within one region of the particle, while the remaining domain remains free of payload, with analogous designs enabling the incorporation of multiple agents. Particle formation is achieved through controlled phase separation of partially miscible solvent systems, leading to the generation of compositionally distinct droplets using microfluidic flow-focusing techniques.

#### 6.1.5. Phase Separation

Internal phase separation has emerged as a broadly applicable strategy for the large-scale fabrication of polymeric Janus and multicompartment particles. This approach relies on the controlled demixing of polymer-containing droplets during solvent removal. Typically, a polymer is dissolved in a mixture composed of a volatile good solvent and a less volatile nonsolvent. The solvent composition is adjusted so that the polymer remains only marginally soluble. This organic phase is subsequently emulsified in an aqueous medium containing stabilizers, generating oil-in-water droplets that serve as confined microreactors.

As the volatile solvent evaporates, the internal composition of each droplet progressively shifts, reducing polymer solubility and triggering phase separation within the droplet. Polymer-enriched domains nucleate and evolve, and their subsequent migration and coalescence are governed by interfacial energy minimization. Ultimately, the system adopts a morphology that corresponds to the lowest total interfacial free energy between the polymer-rich phase, the remaining solvent phase, and the surrounding aqueous environment. Depending on the balance of interfacial tensions, a variety of equilibrium structures can be obtained, including core–shell particles, partially engulfed “acorn-like” geometries, or completely separated domains within a single particle.

The final particle architecture is highly sensitive to formulation parameters such as solvent choice, surfactant type, and concentration. For instance, variations in stabilizer chemistry can shift the preferred morphology from encapsulated structures to anisotropic composite particles. In some systems, solvent evaporation may also generate porous or foamed membranes due to the entrapment and subsequent removal of secondary liquid inclusions. Beyond single-polymer systems, multiphase particles composed of two immiscible polymers can be prepared either by sequential polymerization around preformed seeds or through co-dissolution followed by phase separation during solvent extraction. Adjusting solvent quality and surfactant content enables control over structures ranging from spherical composites to dimpled or partially segregated configurations [99].

A principal advantage of phase separation-based methods lies in their operational simplicity and scalability. The reliance on emulsion processing makes it possible to generate substantial quantities of particles with sizes spanning from the submicron regime to several micrometers. Moreover, diverse morphologies can be accessed without complex equipment or multistep surface modification procedures.

Despite these benefits, important challenges remain. Precise control over particle size distribution and internal structure can be difficult, particularly in conventional emulsion polymerization systems where droplet stability and solvent removal rates influence morphology evolution. In addition, alternative approaches based on block copolymer self-assembly often produce Janus-type micelles only at low concentrations, limiting productivity. Addressing these limitations is essential for advancing phase separation strategies toward more robust and industrially viable manufacturing processes [86].

### 6.2. Janus Nanoparticle Characterization

The identification and validation of JNPs constitute a significant analytical challenge due to their intrinsic anisotropy, which arises from the coexistence of at least two spatially distinct domains with different chemical compositions, surface polarities, or functionalities. Unlike conventional isotropic nanoparticles, the reliable characterization of JNPs requires a multimodal strategy that combines direct morphological visualization, spatially resolved chemical analysis, and the assessment of dynamic physicochemical behavior.

To confirm the anisotropic (“two-faced”) architecture of JNPs, transmission electron microscopy (TEM) and scanning electron microscopy (SEM) are among the most critical and widely employed techniques. TEM has been extensively used to directly visualize asymmetric morphologies such as bullet- or dumbbell-like structures, enabling the con-459 firmation of phase separation between metallic domains (e.g., Ag or Fe_3_O_4_) and silica components [100,101]. In contrast, SEM is particularly useful for examining particles partially embedded in paraffin wax or polymeric masks, thereby validating the effectiveness of Pickering emulsion-based strategies for toposelective surface modification [20,102].

Spatially resolved chemical characterization is equally essential for demonstrating functional compartmentalization. Elemental mapping by energy-dispersive X-ray spectroscopy (EDX) is the most commonly applied approach to reveal the asymmetric distribution of elements such as silicon, gold, silver, or iron across the distinct hemispheres of JNPs. Complementarily, Fourier transform infrared (FT-IR) and nuclear magnetic resonance (NMR) spectroscopies are routinely employed to verify selective ligand immobilization, polymer growth, and the successful execution of surface reactions, including click chemistry and other covalent functionalization strategies [17,103,104].

Beyond structural and compositional analyses, surface properties and functional behavior are commonly evaluated using nitrogen adsorption techniques (BET/BJH) to quantify surface area and porosity, as well as dynamic light scattering (DLS) and zeta potential measurements to assess colloidal stability and surface charge evolution following chemical modification or exposure to external stimuli, such as those associated with the tumor microenvironment. For more complex platforms, including enzyme-powered nanomotors [102], nanoparticle tracking analysis (NTA) and confocal microscopy are indispensable tools for quantifying propulsion velocity, diffusion coefficients, and real-time, spatially controlled drug release within cancer cells.

Table 4 summarizes the most commonly employed physicochemical characterization techniques for the identification and comprehensive analysis of JNPs used as DOX release systems.

## 7. Reported Janus Nanoparticles as Doxorubicin Delivery Systems

Within the broad landscape of nanocarrier-based chemotherapy, JNPs have attracted particular attention as engineered platforms for DOX delivery, owing to their capacity to spatially segregate functionalities within a single particle. Rather than reiterating their general advantages, this section focuses on how Janus architectures have been specifically exploited to address DOX-related challenges, including controlled release, selective targeting, and combination therapy.

The asymmetric structure of JNPs enables the compartmentalization of DOX within a defined domain, while the opposing face can be independently engineered for targeting, stimulus responsiveness, or auxiliary therapeutic functions. This spatial separation is especially advantageous for DOX, whose amphiphilic nature and dose-dependent toxicity demand precise control over loading and release. By integrating chemically distinct environments within one carrier, JNPs overcome limitations associated with conventional isotropic systems, particularly in accommodating both hydrophobic and hydrophilic components relevant to DOX-based therapies.

Furthermore, JNPs designs allow fine control over particle size, shape, and interfacial chemistry, parameters known to influence circulation behavior, cellular uptake, and intracellular trafficking. Several studies have demonstrated that complex Janus geometries can reduce nonspecific macrophage uptake, thereby enhancing circulation persistence and tumor accumulation. Table 5, Table 6 and Table 7 show the most highlighted works performed during the last years, regarding the use of JNPs as DOX delivery system for cancer treatment.

Prior to 2012, reports explicitly describing JNPs as delivery systems for DOX in cancer therapy appear to be limited in the mainstream indexed literature. While a substantial body of work on DOX-based nanomedicine existed during this period, the systematic use of well-defined JNPs architectures for DOX delivery became more evident in the literature from 2012 onward, coinciding with the emergence of several frequently cited primary studies.

The development of Janus nanoparticles represents a significant shift from traditional isotropic drug carriers toward sophisticated, compartmentalized systems that address the complexities of cancer therapy. The following sections provide a critical analysis of the synthesis, architectural advantages, biological relevance, and translational potential of these nanoplatforms.

### 7.1. Dominant Synthesis Strategies and Structural Anisotropy

The literature identify three primary synthesis pathways that dictate the functional compartmentalization and anisotropy of JNPs. The Pickering emulsion interface method is the most prevalent strategy for creating distinct inorganic–organic or inorganic–inorganic faces, typically using paraffin wax to mask one hemisphere of a nanoparticle while the other remains available for toposelective modification [20,22,102]. In contrast, inorganic hybrids such as silver-mesoporous silica or upconverting-magnetic systems often rely on anisotropic or epitaxial growth, where a secondary phase nucleates on a specific site of a seed crystal, resulting in uniform “bullet” or “dumbbell” shapes [100,101,105]. Polymeric systems frequently employ fluidic nanoprecipitation or click chemistry to produce biphasic architectures that can accommodate payloads with disparate solubilities in separate internal compartments [82,103]. These synthesis choices directly influence “hardware” capabilities, where Pickering emulsions provide precise separation for attaching different enzymes or targeting ligands, and anisotropic growth allows for independent mesoporous storage spaces with varied pore sizes or hydrophobicity levels within a single rigid framework [106].

**Table 5:** Janus nanoparticles as doxorubicin delivery system for cancer treatment (Part 1).

Janus Nanoparticles (JNPs)	Synthesis Technique and JNPs Properties	Drug	Type of Cancer	Key Results	Ref.
Polymeric PLGA (PLA/PGA)	Fluidic nanoprecipitation system (FNPS): 305 nm size; dual-compartment structure for hydrophobic and hydrophilic codelivery	DOX + Paclitaxel (PTX)	Not specified (proof-of-concept anticancer drug delivery platform)	80% PTX and 15% DOX encapsulation efficiencies; distinct burst release of PTX confirming the Janus architecture	[82], 2012.
PEGylated Taxol/PLGA	Co-assembly of PEGylated Taxol and PLGA–PEG–PLGA copolymer: 80–200 nm size; hydrophobic core; high colloidal stability	DOX + Taxol (as a prodrug)	Breast cancer (4T1)	Relative tumor size of 30.8% compared to control; 50-h sustained plasma drug concentration	[107], 2013.
Polystyrene/Fe_3_O_4_ @SiO_2_	Combined miniemulsion and sol–gel reaction: 313 nm size; folic acid targeting; pH-cleavable hydrazone linker	DOX	Breast cancer (MDA-MB-231)	82.6% drug release at pH 5.0; 4-fold lower ${\mathrm{IC}}_{50}$ for targeted particles compared to non-targeted ones	[16], 2013.
PLGA/Precirol	Modified W1/O/W2 double emulsion solvent evaporation: “ice cream cone” shaped (polymer head/lipid tail); 155 and 450 nm sizes; zeta potential of −15.22 mV; biodegradable and biphasic	DOX + Curcumin	Lung cancer (A549 human lung adenocarcinoma)	90% cancer cell death in vitro; near-complete tumor suppression in vivo via inhalation; 24 h lung retention for 450 nm particles; recovery of body weight during treatment	[108], 2014.
Upconverting nanoparticles (NaGd F_4_:Yb,Tm @NaGdF_4_) @SiO_2_@meso-porous silica shell/periodic mesoporous organosilica	Anisotropic island nucleation and growth: 300 nm size; dual independent mesopores (2.1 nm and 3.5–5.5 nm); high surface area (1290 m^2^/g); bimodal hydrophilicity/ hydrophobicity	DOX + PTX	Cervical cancer (HeLa cell line)	>50% cancer cell killing efficiency with bimodal (heat + NIR) trigger compared to 25% with single trigger; high biocompatibility (cell viability >90% at 500 μg/mL)	[106], 2014.
Mesoporous silica/Au	Pickering emulsion template method: 130 nm size; pH/redox-responsive; real-time FRET and SERS monitoring	DOX + 6-mercapto-purine (6-MP)	Cervical (HeLa) and breast (MDA-MB-231) cancer	Enhanced synergistic cytotoxicity; 1.96% DOX and 1.18% 6-MP loading by weight; successful real-time monitoring of dual release	[109], 2016.
Ag/Mesoporous silica	Modified sol–gel process (anisotropic growth): −300 nm length; bullet-like shape; SERS-traceable	DOX	Liver (HepG2), lung (A549), and breast (MCF-7)	63.9% encapsulation efficiency; >40% drug release at pH 5.5 within 24 h; selective cytotoxicity against tumor cells	[100], 2016.

**Table 6:** Janus nanoparticles as doxorubicin delivery system for cancer treatment (Part 2).

Janus Nanoparticles (JNPs)	Synthesis Technique and JNPs Properties	Drug	Type of Cancer	Key Results	Ref.
mPEGylated dendron/PVGLIG-DOX	CuAAC click chemistry yielding: 107 nm size; compact self-assembled nanoparticles; near-neutral surface charge; MMP-2/9-responsive PVGLIG linker	DOX	Breast cancer	4.0 wt% DOX loading; enhanced cytotoxicity in MMP-2 environments; similar antitumor efficacy to free DOX but with significantly reduced cardiotoxicity	[12], 2016.
Magnetic Fe_3_O_4_/ Mesoporous silica	High-temperature hydrolysis and sol–gel fabrication: “nanobullet” shaped; Fe_3_O_4_ (100 nm) and mesoporous SiO_2_ body (200 nm), PEGylated and pH-responsive	DOX	Liver cancer (Hepatocellular carcinoma)	61 emu/g saturation magnetization; strong tumor growth inhibition with reduced systemic toxicity; pH-triggered drug release and magnetically enhanced cellular uptake	[100], 2016.
PDA/mesoporous CaP hollow	Island nucleation and anisotropic growth on PAA templates: −150 nm size; hollow cavity; PEG-ICG for photoacoustic (PA) imaging	DOX	Hepatocellular carcinoma (HepG2)	96.2% tumor suppression in vivo; 94.5% drug release (pH 5.0 + NIR); 92% loading efficiency	[13], 2017.
Au nanostar/MSNP	Pickering emulsion interface method: −188 nm size; NIR-responsive gold side; pH-sensitive MSNP gatekeepers	DOX	Cervical cancer (HeLa)	80% drug release (60 min NIR); 15-fold increase in intracellular drug fluorescence after laser irradiation	[17], 2019.
Au/Enzyme-powered mesoporous silica	Mask-protecting assisted site-selective modification: −121 nm size; bi-enzymatic cascade (Invertase/GOx); pH-sensitive gates	DOX	Cervical cancer (HeLa)	On-command drug release triggered by glucose or sucrose; resulting in up to 66% cell death	[110], 2019.
HA/Conjugated magnetic mesoporous silica	Modified sol–gel approach: −300 nm length; CD44 active targeting; dual-drug loading	DOX + Berberine (BER)	Hepatocellular carcinoma	58.8% DOX and 54.2% BER loading; 48.1% apoptotic rate; inhibition of tumor repopulation	[111], 2019.
HA/DMMA-functionalized mesoporous silica	Pickering emulsion interface method: −110 nm size; HA targeting; pH-triggered charge reversal	DOX	Lung cancer (A549)	69.5% encapsulation efficiency; 70% drug release at pH 5.3; 70% survival rate (>40 days) in tumor-bearing mice	[20], 2019.
PMMA/Fe_3_O_4_/ PAA brushes	ARGET-ATRP and in situ co-precipitation: −51 nm size; amphiphilic brushes; superparamagnetic; MRI-active	DOX	Breast cancer (4T1)	89.75% loading capacity; superior synergistic tumor inhibition (0.14 g avg. weight vs. 0.886 g control)	[24], 2020.

**Table 7:** Janus nanoparticles as doxorubicin delivery system for cancer treatment (Part 3).

Janus Nanoparticles (JNPs)	Synthesis Technique and JNPs Properties	Drug	Type of Cancer	Key Results	Ref.
Au/Mesoporous silica	Pickering emulsion method: 113 nm size; pH/NIR-responsive; PTX loaded via β-CD	DOX + PTX	Liver (SMMC-7721) and lung (LLC) cancer	73.5% DOX release at pH 5.0; significant in vivo synergy resulting in 96.5% tumor suppression	[22], 2020.
Polymeric PEG/PCL	Azide-alkyne click chemistry and ATRP: −124 nm size; EpCAM-aptamer targeting	DOX	Colorectal adenocarcinoma (HT-29)	74% encapsulation efficiency; 14.8% loading capacity; high toxicity (${\mathrm{IC}}_{50}$ = 7 μM) at acidic pH	[103], 2020.
Amphiphilic Au nanorod/ZIF-8	Facile method involving epitaxial growth; 100 nm size; LA-modified for ASGP-R active targeting	DOX + Sorafenib (SF)	Hepatocellular carcinoma (recurrence)	68% DOX loading efficiency; improved survival and significant anti-recurrence efficacy in vivo	[112], 2022.
CoFe_2_O_4_ balls/ Mesoporous silica rods	Interfacial energy-mediated anisotropic growth: −375 nm length; microwave-responsive	DOX	Breast, cervical, and lung cancer	73% drug release under microwave at pH 5.3; −87% tumor cell death (in MCF-7)	[113], 2024.
Biphasic PCL/PLGA	Double emulsion (W1/O/W2) technique: −micron; dual-drug loaded; 3D bioprinted scaffold	DOX + Curcumin	General cancer therapy	Sequential sustained release over 14 days; enhanced apoptotic response via drug synergy	[114], 2024.
Ir/Mesoporous silica	Partial surface masking with toposelective modification: −109 nm size; enzyme-propelled	DOX	Cervical cancer (HeLa)	83–85% cell death; autonomous motion with an effective diffusion coefficient of 9.8 μm^2^/s	[102], 2024.
Upconverting nanoparticles (NaY_0.8_F_4_:Yb_0.195_, Tm_0.005_)/ Magnetic Fe_3_O_4_	Thermal decomposition and epitaxial growth: −295 nm size; NIR/UV/ pH-responsive	DOX	Breast cancer (MCF-7)	70% loading efficiency; 22% drug release (pH 6.0 + NIR); cell viability reduced to 26%	[105], 2025.
Asymmetric Ag/Mesoporous silica	Surfactant-templated sol–gel approach: −285 nm size; dumbbell-shaped; aptamer targeting	DOX + antisense oligonucleotides	Multidrug-resistant breast cancer	89% cell death in resistant cells; 80% reduction in P-gp expression; 85.2% encapsulation	[101], 2025.
PAMAM/Mesoporous silica	Partial surface masking with paraffin wax: −102 nm size; anisotropic organic–inorganic nanoparticles on opposite faces	DOX	HeLa cancer cells	Enzyme-controlled drug delivery platform; 43% synthesis yield; successful site-selective assembly of enzyme sensing units; overcomes metallic catalytic limitations decomposition	[104], 2025.

### 7.2. Architecture, Drug Loading, and Release Behavior

The architectural complexity of JNPs is fundamentally designed to implement a “separate rooms” concept for incompatible payloads. Hydrophilic agents like DOX are typically sequestered within mesoporous silica bodies or polymer heads, while hydrophobic drugs such as paclitaxel or sorafenib are loaded into lipid segments, hydrophobic cores, or onto gold surfaces modified with β-cyclodextrin [22,108,114]. Release behavior is governed by sophisticated gating mechanisms tailored to the nanoparticle architecture, transitioning from simple pH-responsive linkers that cleave in acidic environments to active, autonomous nanomachines [16,100]. Advanced designs now utilize enzymatic cascades or near-infrared light to trigger the dissociation of supramolecular gatekeepers [17,110]. Notably, recent “nanomotor” designs use the catalytic decomposition of chemical fuel at a specific nanoparticle face to achieve autonomous propulsion, which simultaneously lowers the local pH to trigger drug release, effectively using the Janus architecture to link physical motion with therapeutic delivery [102].

### 7.3. Synergistic Co-Delivery and Toxicity Mitigation

The combination of DOX with complementary therapeutics in JNPs systems significantly enhances anticancer efficacy while mitigating the drug’s systemic toxicity. The anisotropic architecture of JNPs enables the co-delivery of agents with different solubilities, promoting synergistic therapeutic effects. For example, a dual-compartment JNPs system co-delivering DOX and paclitaxel (PTX) achieved over 50% cell death under bimodal activation, nearly doubling the efficacy of single-trigger systems [106]. Similarly, co-delivery of DOX and curcumin resulted in ∼90% cancer cell destruction in vitro and near-complete tumor suppression in vivo [108]. Importantly, these systems can overcome multidrug resistance (MDR) and reduce recurrence. An 89% cell death rate was reported in resistant breast cancer models using JNPs co-delivering DOX and antisense oligonucleotides targeting P-gp efflux pumps [101], while co-delivery with berberine (BER) suppressed tumor repopulation pathways in hepatocellular carcinoma [18]. In addition to improved efficacy, JNPs reduce DOX-associated cardiotoxicity, as treated models show normal cardiac histology compared to the damage observed with free DOX [12]. This is largely attributed to pH-responsive designs that prevent premature release at physiological pH (7.4) and trigger drug delivery in the acidic tumor microenvironment [102,112]. Consequently, treated animals maintain stable body weight and healthy organs, unlike those receiving free DOX. Finally, targeted co-delivery systems, such as DOX and sorafenib (SF), have demonstrated strong efficacy in preventing postoperative liver cancer recurrence without inducing systemic toxicity [112].

### 7.4. Janus Design and the Tumor Microenvironment

JNPs design is increasingly correlated with specific vulnerabilities of the tumor microenvironment to overcome biological barriers. Targeted designs exploit TME-specific overexpressions through ligands like MUC-1 aptamers for breast cancer, lactobionic acid for liver-specific receptors, and hyaluronic acid for CD44-rich environments [18,101,112]. To combat multidrug resistance, Janus platforms integrate antisense oligonucleotides that downregulate efflux pumps while simultaneously delivering chemotherapeutics, achieved through structural anisotropy that prevents interference between the genetic and chemical components [101]. Furthermore, designs address the TME aftermath of chemotherapy, such as tumor repopulation, by co-delivering inhibitors like berberine to suppress caspase-mediated signaling pathways that would otherwise stimulate the regrowth of residual cancer cells [18]. Systems also utilize the acidic pH of the TME to trigger charge reversal, switching from a negative surface charge that prolongs blood circulation to a positive charge that enhances cellular uptake upon arrival at the tumor site [20,113].

### 7.5. Quantitative Outcomes and Lack of Standardization

A comparative analysis of quantitative outcomes reveals high therapeutic performance but highlights significant reporting variability and a lack of standardization across the field. Reported doxorubicin loading efficiencies vary widely, from as low as 4.0 wt% in peptide-dendron conjugates to as high as 92% in hollow Janus structures [12,13]. Therapeutic success is also measured through diverse metrics, ranging from a 30.8% relative tumor size to 96.5% tumor suppression in optimized in vivo models [22,107]. This variability in reporting metrics—where some studies emphasize ${\mathrm{IC}}_{50}$ reductions and others prioritize survival days or apoptotic rates—creates a significant knowledge gap that hinders cross-platform comparisons and meta-analysis [16,20]. Furthermore, while in vitro cell death often exceeds 80%, the translation to in vivo efficacy is less uniform, raising questions about reproducibility when transitioning to more complex biological systems [101,102].

## 8. Conclusions and Outlook

The development of JNPs represents a significant shift from traditional isotropic drug carriers toward sophisticated, compartmentalized systems that address the complexities of cancer therapy. Their inherent structural asymmetry enables a “separate rooms” concept, allowing for the co-delivery of DOX with incompatible payloads while maintaining precise control over release kinetics. Evidence from the recent literature suggests that JNPs can effectively mitigate DOX-induced cardiotoxicity by enhancing tumor-site accumulation and utilizing site-specific triggers, such as the acidic tumor microenvironment or enzymatic cascades.

However, several hurdles remain for their clinical adoption. The fabrication complexity of many JNP architectures continues to limit scalability and production throughput. Furthermore, there is a notable lack of standardization in reporting metrics, which hinders cross-platform comparisons and meta-analysis of therapeutic efficacy. Future research should focus on developing robust, industrially viable manufacturing processes and standardized characterization protocols. Ultimately, by bridging the gap between advanced material engineering and biological precision, JNPs hold the potential to transform medical practice through precision oncology, providing more effective and less toxic treatments for patients worldwide.

## Figures and Tables

**Table 1:** Most commonly diagnosed cancers globally in 2025 (GCO) [40].

Cancer Type	New Cases (Millions)	Percentage of All New Cases (%)
Lung cancer	2.7	7.3
Female breast cancer	2.5	6.9
Colorectal cancer	2.1	6.1
Prostate cancer	1.6	7.0
Non-melanoma skin cancer	1.3	6.6
Stomach cancer	1.0	7.0
Liver cancer	0.9	6.7

**Table 2:** Doxorubicin (DOX) release systems based on conventional nanoparticles (NPs) versus Janus nanoparticles (JNPs).

Aspect	Conventional Nanoparticles	Janus Nanoparticles
Structural design	Isotropic systems (liposomes, polymeric NPs, micelles).	Anisotropic, multi-compartment architectures.
DOX loading strategy	Encapsulation or conjugation within a single matrix.	Compartmentalized loading; DOX isolated from other cargos or functions.
Release mechanism	Diffusion- or degradation-controlled release, often showing burst release.	Compartment-specific, programmable, and delayed release.
Stimuli responsiveness	Typically responsive to a single stimulus (e.g., pH or enzymatic cleavage).	Multi-stimuli responsiveness (e.g., pH, NIR, magnetic fields, enzymatic triggers).
Combination therapy with DOX	Requires co-encapsulation, which may lead to unsynchronized pharmacokinetics.	Intrinsic spatial separation enables synchronized multi-drug or chemo-physical therapy.
Targeting strategy	Uniform surface functionalization.	Independent functionalization of each compartment.
Effect on DOX cardiotoxicity	Reduced compared to free DOX but still present.	Further reduction reported due to controlled release and targeted delivery.
Macrophage uptake	Typically high for spherical particles.	Reduced uptake reported due to anisotropic geometry.
Manufacturing complexity	Relatively simple and scalable fabrication.	More complex fabrication and quality control requirements.
Clinical translation status	Several clinically approved systems exist.	Currently at the preclinical stage; no Janus DOX systems approved yet.
Main advantages	Regulatory maturity and established safety profiles.	Superior control over DOX release and multifunctionality.
Main limitations	Limited control over combination therapy and release kinetics.	Challenges in scalability, cost, and regulatory approval.

**Table 3:** Comparison of synthesis methods for Janus nanoparticles (JNPs).

Method	Description	Advantages	Limitations
Self-assembly	Phase separation of block copolymers due to thermodynamic incompatibility.	Scalable production; reaches the nano-scale (<100 nm).	Complex molecular design; difficult to orient.
Masking	One hemisphere is protected at an interface (solid/liquid) while the other is modified.	Precise control over patch area; high chemical versatility.	Low yield; difficult to scale up for industrial use.
Glancing angle deposition (GLAD)	Physical vapor deposition on a particle bed at a specific glancing angle.	Clean (solvent-free); precise metallic coating.	Requires high vacuum; limited to surface coatings.
Microfluidics	Use of laminar flows of immiscible polymers followed by in-situ polymerization.	Excellent monodispersity; total control over complex shapes.	High equipment cost; limited to micro-scale particles.
Phase separation	Emulsification of polymer mixtures followed by solvent evaporation.	Simple and cost-effective; high throughput.	Less uniform size distribution compared to microfluidics.

**Table 4:** Physicochemical techniques employed for the identification and characterization of Janus nanoparticles (JNPs) used as doxorubicin (DOX) release systems.

Category	Key Techniques	Information Obtained
Morphology and structure	TEM/HR-TEM/STEM	Direct visualization of asymmetric architectures (e.g., snowman, bullet, dumbbell, or ice-cream cone shapes) and confirmation of phase separation between nanoparticle compartments.
	SEM/FE-SEM	Analysis of surface morphology and roughness after modification; observation of nanoparticles partially embedded in paraffin wax masks during masking-based synthesis.
	AFM	Nanoscale surface topography and detection of mechanical variations or surface indentations depending on the polymer or coating layer.
	XRD/PXRD (small and wide angle)	Identification of crystalline phases of individual components (e.g., Au, Ag, Fe_3_O_4_, upconverting nanoparticles) and ordering of mesoporous structures such as MCM-41.
Chemical composition	EDX/EDS/Elemental mapping	Determination of elemental distribution and verification of spatial compartmentalization within Janus architectures.
	NMR (^1^H, ^13^C)	Verification of polymer synthesis, photolabile ligands, and efficiency of conjugation reactions on nanoparticle surfaces.
	MALDI-TOF/SEC-MALS	Determination of molecular weight and confirmation of dendrimer–drug or polymer conjugate formation.
Surface properties	$N_{2}$ adsorption (BET/BJH)	Measurement of surface area, pore volume, and pore size distribution; monitoring structural changes after DOX loading.
	Hydrodynamic size (DLS)	Determination of nanoparticle diameter in suspension and evaluation of colloidal stability or aggregation behavior.
	Zeta potential	Surface charge analysis to confirm chemical modifications and pH-triggered charge reversal.
Functional properties	UV-Vis–NIR/PL	Optical absorption of targeting ligands and photoluminescence of UCNPs; monitoring drug release behavior.
	Magnetometry (VSM)	Hysteresis curves and magnetic saturation used to validate superparamagnetic properties of Fe_3_O_4_ or CoFe_2_O_4_ cores.
	Thermogravimetric analysis (TGA)	Quantification of organic components, ligands, and drugs thermally anchored to the nanoparticle surface.
Biological validation	Confocal/Fluorescence microscopy	Visualization of cellular internalization and differential loading using Janus-specific fluorescent probes.
	Nanoparticle tracking analysis (NTA)	Measurement of displacement velocity and diffusion coefficients in enzyme-powered nanomotors.
	Gel electrophoresis	Confirmation of the binding of antisense oligonucleotides or aptamer strands to the nanoparticle surface.

## Data Availability

No new data were created or analyzed in this study. Data sharing is not applicable to this article.
